# Unraveling low-resolution structural data of large biomolecules by constructing atomic models with experiment-targeted parallel cascade selection simulations

**DOI:** 10.1038/srep29360

**Published:** 2016-07-05

**Authors:** Junhui Peng, Zhiyong Zhang

**Affiliations:** 1 Hefei National Laboratory for Physical Science at Microscale and School of Life Sciences, University of Science and Technology of China, Hefei, Anhui 230026, People’s Republic of China

## Abstract

Various low-resolution experimental techniques have gained more and more popularity in obtaining structural information of large biomolecules. In order to interpret the low-resolution structural data properly, one may need to construct an atomic model of the biomolecule by fitting the data using computer simulations. Here we develop, to our knowledge, a new computational tool for such integrative modeling by taking the advantage of an efficient sampling technique called parallel cascade selection (PaCS) simulation. For given low-resolution structural data, this PaCS-Fit method converts it into a scoring function. After an initial simulation starting from a known structure of the biomolecule, the scoring function is used to pick conformations for next cycle of multiple independent simulations. By this iterative screening-after-sampling strategy, the biomolecule may be driven towards a conformation that fits well with the low-resolution data. Our method has been validated using three proteins with small-angle X-ray scattering data and two proteins with electron microscopy data. In all benchmark tests, high-quality atomic models, with generally 1–3 Å from the target structures, are obtained. Since our tool does not need to add any biasing potential in the simulations to deform the structure, any type of low-resolution data can be implemented conveniently.

Biological function of a large biomolecule (protein, DNA, RNA, or complex) relies on its three dimensional structure, which involves conformational changes when performing the function. To better understand the structure-function relationship of the biomolecule, it would be beneficial to determine the structures of all its conformational states. Although X-ray crystallography or solution nuclear magnetic resonance (NMR) is widely used in solving high-resolution structures of biomolecules, it is often difficult to capture all the conformational states of a biomolecule with atomistic details. In this case, some alternative experimental techniques, such as small-angle X-ray scattering (SAXS)^1^, electron microscopy (EM)^2^, paramagnetic relaxation enhancement (PRE)^3^, pseudocontact shifts (PCS)^4^, single-molecule fluorescence resonance energy transfer (smFRET)^5^, and chemical cross-linking with mass spectrometry (CXMS)^6^, could be conducted to obtain structural information of the biomolecule at relatively low-resolution level.

In order to unravel the structural information encoded in the low-resolution data precisely, it is necessary to construct an atomic model (or an ensemble) of the biomolecule that best fits the data. Since low-resolution experimental techniques generally cannot supply sufficient data to determine the atomic structure alone, they have to be aided by computational modeling, such as molecular dynamics (MD) simulations with biomolecular force field. To date, there are various such integrative modeling techniques for the interpretation of different low-resolution structural data^7^,^8^,^9^,^10^,^11^,^12^,^13^,^14^,^15^,^16^,^17^,^18^,^19^,^20^. Many of these methods use a refining-while-sampling strategy, which adds an extra pseudo-energy term based on the given low-resolution data into the molecular mechanics energy function, and the resulting additional forces will deform the biomolecular structure accordingly to fit the experimental data.

This work aims to develop a general simulation tool, which can fit any low-resolution structural data conveniently without modifying the energy function and build the corresponding atomic model of the biomolecule. The idea was from a sampling technique called parallel cascade selection molecular dynamics (PaCS-MD)^21^,^22^. The PaCS-MD method consists of cycles of (1) conformational sampling by multiple independent MD (miMD) simulations and (2) selecting a number of conformations from the miMD trajectories, which are closest to the target structure^21^ or mostly deviate from the average structure (if no target structure is available)^22^, to start the next cycle of miMD simulations. It has been reported that this sampling technique can efficiently enhance conformational transitions of large biomolecules. In our problem of interpreting a given type of low-resolution structural data, we only need to design a scoring function that measures the discrepancy (or similarity) between a simulated conformation and the target experimental data. After each cycle of miMD, we pick a number of conformations with the smallest discrepancy (or highest similarity) to the target data for the next cycle. By this iterative screening-after-sampling strategy, our experiment-targeted PaCS-based method (termed as PaCS-Fit) may allow the biomolecule to approach an atomic model that is consistent to the target low-resolution data.

We have tried our method using two types of low-resolution structural data from SAXS and EM, respectively. The SAXS-targeted PaCS-Fit is tested on three proteins, which are *E. coli* adenylate kinase, hen egg-white lysozyme, and the triple-BRCT-domain of epithelial cell transforming protein 2. The EM-targeted PaCS-Fit is also illustrated by *E. coli* adenylate kinase, and another protein the GroEL monomer. All the results indicate the efficiency of the PaCS-Fit method.

## Results

### SAXS-targeted PaCS-Fit

SAXS has gained its popularity in structural biology over the past decades^23^. Despite low-resolution nature of the one dimensional scattering profile, SAXS may provide overall information like the size and shape of a biomolecule^24^. The scoring function for SAXS-targeted PaCS-Fit is the discrepancy (denoted as χ) between the theoretical scattering profile of a biomolecular conformation and the target SAXS data, computed by equation (1) in Methods.

#### *E. coli* adenylate kinase (AKeco)

It is a 214-residue protein with three domains, which are the CORE domain (residues 1–29, 60–121, and 160–214), the LID domain for ATP-binding (residues 122–159) and the NMP domain for AMP-binding (residues 30–59), respectively. The kinase can catalyze the reaction MgATP + AMP ↔ 2ADP + Mg^2+^ and regulate the concentration of ATP within the cell. During its catalytic cycle, the protein undergoes a large conformational change in that the LID and NMP domains can open or close relative to the CORE domain^25^. Crystal structures of AKeco in both the closed (with an inhibitor Ap5A, PDB entry 1AKE^26^) and the open (PDB entry 4AKE^27^) state have already been solved, which enable us to run PaCS-Fit by choosing one structure as the initial conformation and fitting it to the simulated SAXS data of the other state, in order to test model reproducibility with the simulated data^28^. The procedure to obtain the simulated target SAXS data can be found in Methods.

**Figure 1:**
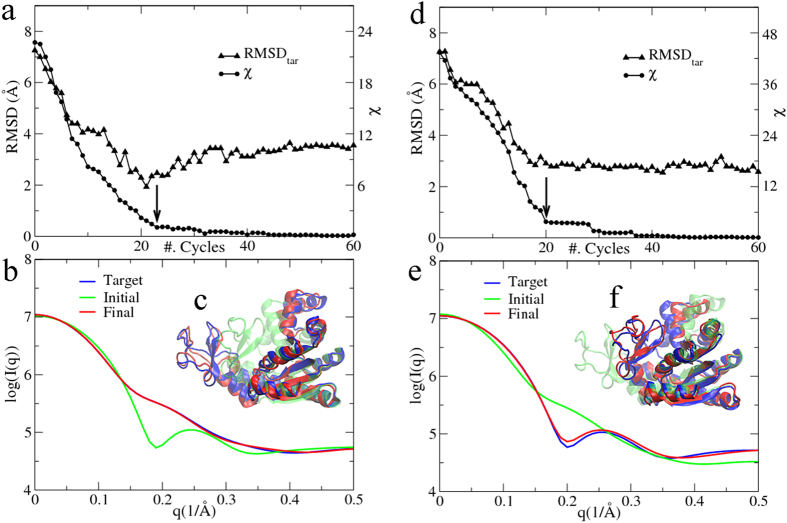
SAXS-targeted PaCS-Fit of AKeco. The left panel shows the fitting from the closed to the open state. (**a**) The minimal χ to the simulated target SAXS profile at each cycle (circles), and the corresponding RMSD_tar_ (up-triangles). In order to avoid overfitting, the final structural model was chosen at the 23^rd^ cycle (indicated by an arrow) since χ becomes saturated after this. (**b**) Simulated SAXS profile of the initial structure (green) and the final model (red), along with the target SAXS data (blue). (**c**) The initial structures (green transparent), the final model (red), and the target structures (blue). The right panel shows PaCS-Fit from the open to the closed state, and the final structural model was chosen at the 20^th^ cycle. All the protein structures were visualized using VMD^56^.

PaCS-Fit was firstly carried out using the simulated SAXS profile of the open AKeco structure as the target, starting from its closed structure. It has been reported that interpretation of low-resolution SAXS data is susceptible to overfitting^8^. In order to illustrate this issue, the PaCS-Fit of AKeco was run for 60 cycles. The minimal χ of each cycle is plotted (Fig. 1a, circles). It can be seen that χ decreases quickly in the first 20 cycles and slowly after the 23^rd^ cycle. Correspondingly we calculated root mean squared deviation (RMSD) of these conformations to the target open structure (RMSD_tar_, Fig. 1a, up-triangles), in order to show the accuracy of the model constructed from the SAXS data. The initial closed structure has a RMSD_tar_ of 7.3 Å. Similar to the χ values, RMSD_tar_ decreases quickly in the first 20 cycles and reaches to the smallest value of 2.0 Å at the 21^st^ cycle. However, it starts to increase afterwards (3.5 Å at the last cycle) although χ continues to decrease slightly, which indicates an overfitting problem.

Since the target atomic structure is not available in a real project using PaCS-Fit, RMSD_tar_ cannot serve as a criterion to pick the final structural model. Other criteria, such as the scoring function itself (here is χ), RMSD to the initial conformation (denoted as RMSD_ini_), and radius of gyration (*R*_*g*_) could be used. We have compared these metrics in the Supplementary Information, and found that it seems reasonable to determine the final conformation to fit the low-resolution SAXS data according to the saturation of χ in order to avoid overfitting. According to this criterion, we pick the conformation at the 23^rd^ cycle as the final model because χ starts to saturate at this point.

The theoretical SAXS profile of the initial closed structure (Fig. 1b, green curve) has a χ = 22.7 against the target data (Fig. 1b, blue curve), whereas that of the final picked structure (Fig. 1b, red curve) is nearly identical to the target one with χ = 1.0. Accordingly the structural model (Fig. 1c, red) looks quite similar to the target structure (Fig. 1c, blue) with a RMSD_tar_ of 2.5 Å. It should be noted that in a conventional MD simulation at the same time scale, the ligand-free AKeco could hardly transit from the closed to the open state. The results indicate that PaCS-Fit can successfully unravel the simulated SAXS data of the open AKeco, and generate an atomic model that is close to the available crystal structure.

Then we tested PaCS-Fit in a reverse process by fitting with the simulated SAXS data of the closed state, starting from the open AKeco structure. The results are shown in Fig. 1, the right panel. Again according to the curves of χ and RMSD_tar_ (Fig. 1d, circles and up-triangles, respectively), it is reasonable to pick the conformation at the 20^th^ cycle as the final model since χ tends to saturate after this point. The theoretical SAXS profile of this model shows a much smaller χ = 3.8 (Fig. 1e, red curve) against the target SAXS data (Fig. 1e, blue curve) than that of the initial structure (Fig. 1e, green curve, χ = 43.4). The final atomic model (Fig. 1f, red) has a RMSD_tar_ of 2.9 Å to the closed structure (Fig. 1f, blue).

Overall, the above results support that PaCS-Fit can work very well for both the close-to-open and open-to-close fitting of AKeco. The saturation point of χ may be used as a criterion to choose the final structural model.

#### Hen egg-white lysozyme (HEWL)

The crystal structure of 129-residue HEWL is available (PDB entry 6LYZ^29^) as the target structure, and its SAXS data was taken from www.bioisis.net (BioIsis ID LYSOZP). We built an atomic model with a RMSD of 6.2 Å to the crystal structure (see Methods), which was used as the initial conformation of PaCS-Fit.

**Figure 2:**
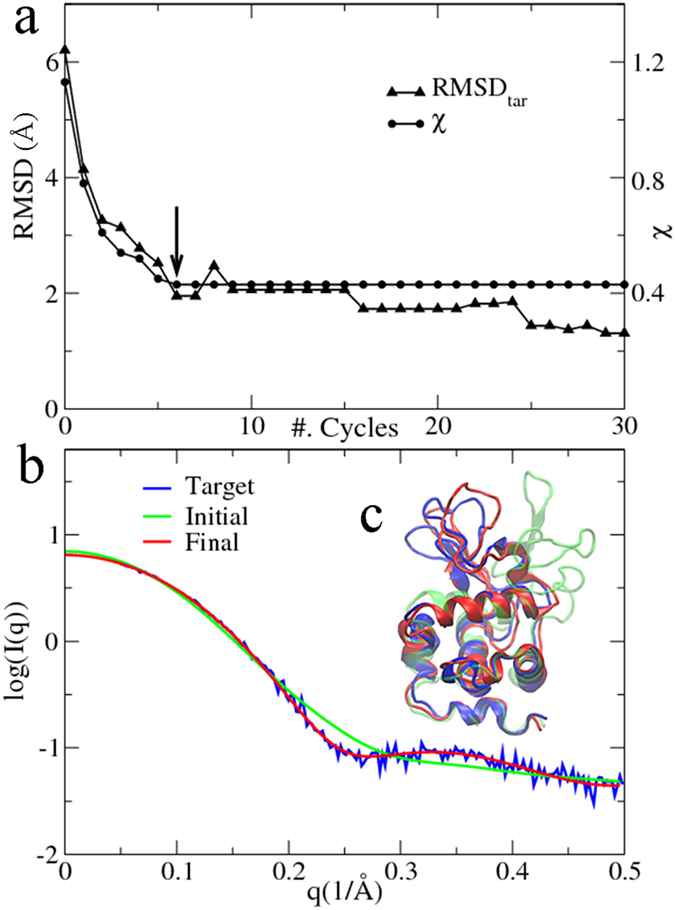
SAXS-targeted PaCS-Fit of HEWL. (**a**) The minimal χ to the experimental target SAXS data at each cycle (circles), and the corresponding RMSD_tar_ (up-triangles). The final structural model was chosen at the 6^th^ cycle (indicated by an arrow). (**b**) Simulated SAXS profiles of the initial structure (green) and the final model (red), along with the experimental target SAXS data (blue). (**c**) The initial structure model (green transparent), the final model (red), and the target structure (blue).

30 cycles of PaCS-Fit simulations were carried out. According to the curve of χ (Fig. 2a, circles), the theoretical SAXS profile of the initial structure (Fig. 2b, green curve) has a χ = 1.1 to the experimental SAXS data (Fig. 2b, blue curve), which decreases quickly during the first five cycles. After this point, χ becomes saturated. Therefore, we pick the conformation at the 6^th^ cycle as the final model to fit the SAXS data with χ = 0.4 (Fig. 2b, red curve). The RMSD between this final structure (Fig. 2c, red) and the target crystal structure of HEWL (Fig. 2c, blue) is only 1.9 Å. From the RMSD_tar_ curve (Fig. 2a, up-triangles), the conformation at the 30^th^ cycle has the smallest RMSD_tar_ of 1.3 Å. Although this conformation would not be chosen based on our criterion, it is fairly to say that the final model still has a reasonable RMSD_tar_ smaller than 2.0 Å.

To estimate precision of the structural model, we performed PaCS-Fit of HEWL for ten times independently, starting from the same initial conformation. The results are shown in the Supplementary Information. The ten final structural models are very similar, which indicate that the models of HEWL built by SAXS-targeted PaCS-Fit should be reliable.

#### Triple-BRCT-domain of epithelial cell transforming protein 2 (ECT2)

The three tandem BRCT domains at the N-terminal of ECT2 play a critical role in regulating cytokinesis^30^, and its crystal structure (residues 22 to 236) has been elucidated (PDB entry 4N40)^31^. However, the experimental SAXS data indicates a discrepancy between the protein structure under crystal packing and that in solution. In this paper, starting from the crystal structure, we ran PaCS-Fit to obtain an atomic model of the protein that reproduces the SAXS data.

**Figure 3:**
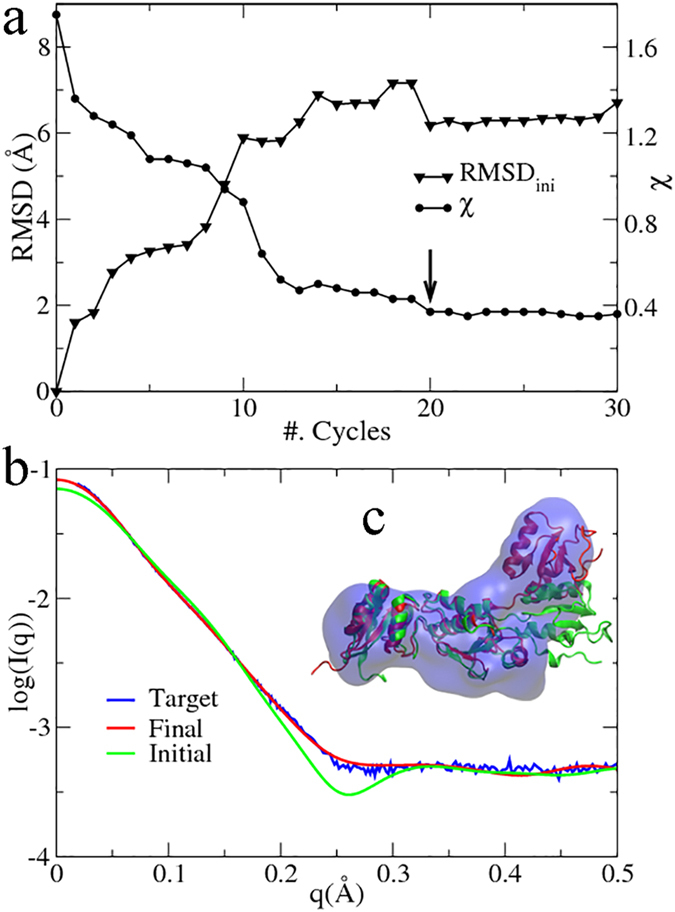
SAXS-targeted PaCS-Fit of the triple-BRCT-domain of ECT2. (**a**) The minimal χ to the experimental SAXS data at each cycle (circles), and the corresponding RMSD_ini_ (down-triangles). The final structural model was chosen at the 20^th^ cycle (indicated by an arrow). (**b**) Simulated SAXS profiles of the initial structure (green) and the final model (red), along with the experimental SAXS data (blue). (**c**) The initial structure (green), the final model (red) and the dummy-residue model (blue transparent) constructed by GASBOR.

It is found that χ does decrease in 30 cycles (Fig. 3a, circles), from the initial value of 1.8 between the theoretical SAXS curve of the crystal structure (Fig. 3b, green curve) and the target data (Fig. 3b, blue curve), and accordingly RMSD_ini_ increases significantly (Fig. 3a, down-triangles). The results suggest that the protein may take a different conformation in solution. We choose the conformation at the 20^th^ cycle as the final structural model since χ tends to saturate after this point, and its theoretical SAXS curve (Fig. 3b, red curve) has a small χ = 0.4 against the experimental data. Meanwhile, RMSD_ini_ seems to be in equilibrium between 6–7 Å. The crystal structure shows a linear arrangement of the three BRCT domains (Fig. 3c, green), whereas a dummy-residue model constructed by GASBOR^32^ (Fig. 3c, blue transparent) indicates that one of the BRCT domains bends towards the other two. In the final model (Fig. 3c, red) with a RMSD_ini_ of 6.2 Å, the third BRCT domain does bend that can match the dummy-residue model very well by SUPCOMB^33^. The PaCS-Fit result is in agreement with the crystallographic data in that temperature factors of the third BRCT domain are higher than those of the first and second one^31^.

For this protein, the target structure is unknown. Therefore, starting from the crystal structure, we carried out ten independent PaCS-Fit, in order to assess the model precision. Although their χ values are all small, and *R*_*g*_ are all consistent to the value estimated from the experimental SAXS data, the ten structural models demonstrate some different conformations (see Supplementary Information). The results may suggest that the SAXS data alone is insufficient to determine a single model of the protein or there exist multiple conformations^28^. More experimental data would be needed to resolve this ambiguity.

### EM-targeted PaCS-Fit

EM is developing rapidly in recent years^34^, which can now be used to determine structures of biomolecular complexes at near-atomic resolution (generally 3–4 Å)^35^. However, many EM structures are still at low-resolution range (>5 Å) since they are much easier to achieve than those high-resolution ones. The scoring function for EM-targeted PaCS-Fit is described as cross-correlation coefficient (CC) between the simulated map of a biomolecular conformation and the target EM map, computed by equation (2) in Methods.

#### AKeco

This protein was also used to test PaCS-Fit using the EM data. The target EM map of AKeco (in either open or closed state) was simulated from its crystal structure using the program pdb2vol in the Situs package^36^. A smoothed Gaussian function with voxel size 1.0 Å was used, and a map at a resolution of 5 Å was generated.

**Figure 4:**
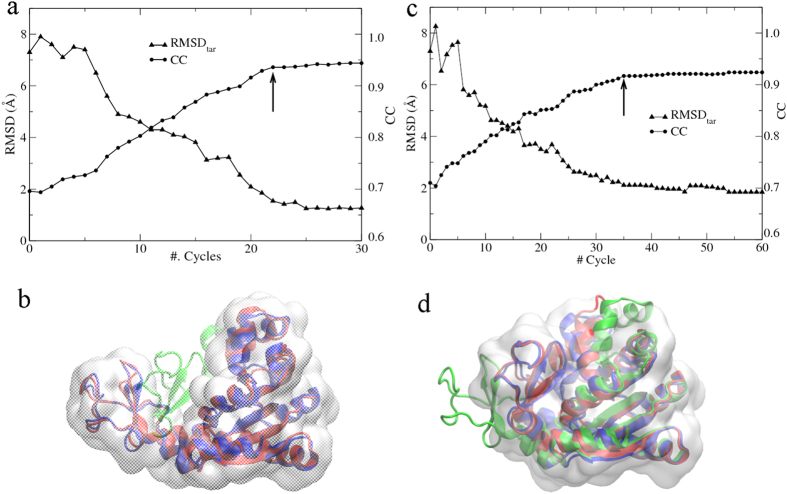
EM-targeted PaCS-Fit of AKeco. The left panel shows the fitting from the closed to the open state. (**a**) The maximal CC value to the simulated target EM map at each cycle (circles), and the corresponding RMSD_tar_ (up-triangles). The final structural model was chosen at the 22^nd^ cycle (indicated by an arrow). (**b**) The initial structure (green), the final model (red), the target structure (blue), and the target EM map (gray). The right panel shows PaCS-Fit from the open to the closed state, and the final structural model was chosen at the 35^th^ cycle.

Firstly, PaCS-Fit from the closed structure to the simulated EM map of the open state was carried out for 30 cycles. The largest CC in each cycle is plotted in Fig. 4a (circles), and the final structural model is still determined according to the saturation of the scoring function. Since the EM data has generally higher resolution than the SAXS data, the overfitting problem in EM-targeted PaCS-Fit is not as serious as that in SAXS-targeted PaCS-Fit. The initial CC value is 0.70, and after 22 cycles, it becomes saturated. Thus, we pick the conformation at the 22^nd^ cycle as the final structure model (Fig. 4b, red), which fits very well with the target EM map (Fig. 4b, gray) with CC = 0.94. According to the RMSD_tar_ (Fig. 4a, up-triangles) to the open AKeco structure (Fig. 4b, blue), this model has a small RMSD_tar_ of 1.5 Å.

Then the reverse fitting from the open structure to the simulated EM map of the closed state was done. It was found that CC did not reach equilibrium during the first 30 cycles, and therefore we extended PaCS-Fit to 60 cycles. From the CC values (Fig. 4c, circles), we pick the conformation at the 35^th^ cycle as the final structural model with CC = 0.92 to the target map (Fig. 4d, gray). The RMSD between this model (Fig. 4d, red) and the target closed structure (Fig. 4d, blue) is 2.1 Å (Fig. 4c, up-triangles).

We have compared the above results with those of SAXS-targeted PaCS-Fit. The final models of AKeco generated by EM-targeted PaCS-Fit have smaller RMSD_tar_ values (Fig. 4a,c, up-triangles) than those generated by SAXS-targeted PaCS-Fit (Fig. 1a,d, up-triangles). The results were also summarized in the Supplementary Table S1. The simulated EM maps of AKeco have resolution of 5 Å that are significantly higher than the SAXS profiles, therefore PaCS-Fit with the EM data may obtain atomic models with better accuracy than PaCS-Fit with the SAXS data.

PaCS-Fit of AKeco targeted by the SAXS and EM data separately may provide a cross validation to assess the accuracy of those structural models, and more details can be found in the Supplementary Information. We also tried a close-to-open PaCS-Fit for the protein by using the SAXS and EM data simultaneously, and the results are shown in the Supplementary Information.

#### GroEL monomer

As a molecular chaperonin found in many bacteria, GroEL is required for proper folding of nascent or stress-denatured polypeptides^37^. The structure of GroEL is a double-ringed tetradecamer that has been investigated by both X-ray crystallography^38^ and EM^39^,^40^. The key of GroEL’s activity is the conformational changes of its monomer between the closed and the open state.

To setup a close-to-open PaCS-Fit, a starting conformation of the monomer at the closed state was extracted from a crystal structure of the 14-mer GroEL complex (PDB entry 1OEL^38^, chain D was selected), and a target EM map at the open state was segmented from an 8.5 Å map of the 14-mer GroEL:ATP complex (EMDB code 2003)^40^. The segmentation was done using the tool^41^ implemented in UCSF Chimera^42^.

**Figure 5:**
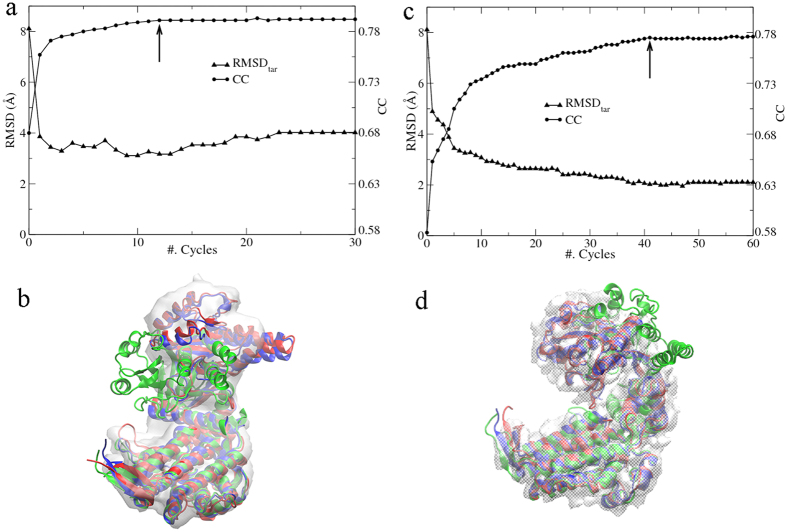
EM-targeted PaCS-Fit of the GroEL monomer. The left panel shows the fitting from the closed to the open state. (**a**) The maximal CC value to the experimental target EM map at each cycle (circles), and the corresponding RMSD_tar_ to an atomic model at the open state constructed by Flex-EM^12^ (up-triangles). The final structural model was chosen at the 12^th^ cycle (indicated by an arrow). (**b**) The initial structure (green), the final model (red), the target open structural model (blue), and the target EM map (gray). The right panel shows PaCS-Fit from the open to the closed state, and the final structural model was chosen at the 41^st^ cycle.

By a rigid-body fitting using the colores program^43^ in the Situs package^36^, the CC value between the initial closed structure and the target EM map of the GroEL monomer is 0.68. During 30 cycles of PaCS-Fit, CC increases to 0.79 and has become saturated after 12 cycles (Fig. 5a, circles). Therefore, we pick the conformation at the 12^th^ cycle as the final structure model (Fig. 5b, red) to fit the target EM map of the open GroEL monomer (Fig. 5b, gray). Clare *et al*. have used the aforementioned 8.5 Å EM map to build an atomic model of the 14-mer GroEL complex (PDB entry 4AB3^40^). Chain D of the model was selected as the target atomic structure here (Fig. 5b, blue), which has a CC = 0.81 with the target EM map. RMSD_tar_ to this structure (Fig. 5a, up-triangles) drops quickly from 8.1 Å to 3.1 Å in the first 9 cycles. After the 13^th^ cycle, the RMSD_tar_ values rise gradually and stabilize at ~4.0 Å after the 23^rd^ cycle. The picked structure model (Fig. 5b, red) has a RMSD_tar_ of 3.2 Å, and its CC value (0.79) is comparable to that of the target structure (0.81).

For the open-to-close PaCS-Fit, the initial open structure of the monomer was the target atomic model in the above close-to-open fitting, and the target EM map at the closed state was segmented from a 4.2 Å map of the 14-subunit GroEL complex^39^. The initial structure has a CC of 0.58 to the target EM map. After 30 cycles of PaCS-Fit simulations, it was found that CC values were not converged, so another follow-up 30 cycles were carried out (Fig. 5c, circles). The final model (Fig. 5d, red) was picked at the 41^st^ cycle. The CC value of this model reaches to 0.78, which is close to that (0.80) between the target atomic structure (chain D of PDB 1OEL^38^, Fig. 5d, blue) and the target map (Fig. 5d, gray). RMSD_tar_ of the final model is as small as 2.0 Å (Fig. 5c, up-triangles).

## Discussion

In this paper, we have presented a new computational tool called PaCS-Fit that can construct an atomic model of a large biomolecule from any type of the low-resolution structural data. Starting from an already known structure of the biomolecule, the method tends to predict a different conformation that satisfies the target low-resolution data. Instead of adding a biasing potential to deform the structure, we take the advantage of multiple independent simulations to sample the conformational space of the biomolecule freely, and then use an iterative screening-after-sampling procedure to drive the conformation towards the target. The results of several benchmark studies have demonstrated that, when the scoring function starts to saturate, our approach is generally able to construct atomic models within 3 Å of the target structure. Such accuracy would be good enough to interpret the low-resolution data. Furthermore, the method may elucidate structural dynamics of the biomolecule and build a transition pathway as by the original PaCS-MD method^21^,^22^.

There are various modeling techniques integrating either SAXS^44^ or EM data^45^, and some of them have also used AKeco and the GroEL monomer to test their methods. Our SAXS-targeted PaCS-Fit of AKeco can obtain atomic models with RMSD_tar_ of 2–3 Å (Fig. 1), which are close to those constructed by other methods^7^,^8^. For the close-to-open fitting of AKeco, EM-targeted PaCS-Fit can obtain an atomic model with RMSD_tar_ = 1.5 Å and CC = 0.94 (Fig. 4, the left panel) while models built by other methods had RMSD_tar_ from 0.7 to 1.6 Å and CC from 0.99 to 0.91^11^,^13^,^14^,^15^. For the close-to-open fitting of the GroEL monomer, EM-targeted PaCS-Fit has obtained an atomic model with CC = 0.79 (Fig. 5, the left panel) while other methods could get models with CC from 0.67 to 0.81^16^,^40^. For the open-to-close fitting of the GroEL monomer, the atomic model constructed by EM-targeted PaCS-Fit has a RMSD_tar_ = 2.0 Å and CC = 0.78 (Fig. 5, the right panel), which is similar to that built by Flex-EM^12^ (RMSD_tar_ = 1.9 Å and CC = 0.75). These results of PaCS-Fit do support that our tool is as good as other integrative modeling methods.

The major advantage of PaCS-Fit is that no biased energy term is used, and therefore it is not required to modify the complicated simulation (such as MD) program. This would enable us to integrate any low-resolution structural data into modeling easily. For a given type of the data, the most important thing of PaCS-Fit is to translate it into a scoring function, which could be done by either calling an existing program^46^,^47^ or writing a short one. The remaining issues are just to prepare a couple of relatively simple scripts that select conformations, restart the next cycle of simulations, and so on. The computational cost of our method (see Supplementary Information) may be larger than other methods since PaCS-Fit consists of a series of multiple independent simulations. However, PaCS-Fit is very suitable for massive parallel computing. On the other hand, for a very large biomolecular system, conventional MD can be replaced by enhanced sampling techniques or coarse-grained simulations in order to accelerate conformational sampling.

Applications of PaCS-Fit in this paper have used χ (equation (1) in Methods) as the scoring function for the SAXS data and CC (equation (2) in Methods) for the EM data. We could also try other alternative functions for SAXS^7^,^48^,^49^ or EM^50^,^51^, in order to compare their fitting efficiency when the target data is the same. We believe that the PaCS-Fit method is not only restricted to SAXS or EM data, but also can be used to any other experimental data^3^,^4^,^5^,^6^ as long as a proper scoring function is obtained. Furthermore, the scoring function could be a combination of different sources of data. We have successfully done PaCS-Fit of AKeco targeted by the SAXS and EM data simultaneously (see Supplementary Information). By integrating structural information of binding interfaces and global shape obtained from SAXS or EM, PaCS-Fit may be able to build a structural model of a biomolecular complex from its components. The above would be future scopes of our research.

## Methods

The idea of PaCS-Fit is to run a series of independent simulations, and finally obtain a conformation of the biomolecule that can best fit the given low-resolution structural data. To achieve this goal, we need simulation methods to sample various conformations of the biomolecule, scoring functions to describe difference (or similarity) between any simulated conformation and the target low-resolution data. These issues will be introduced in this section.

### General work flow of PaCS-Fit

**Figure 6:**
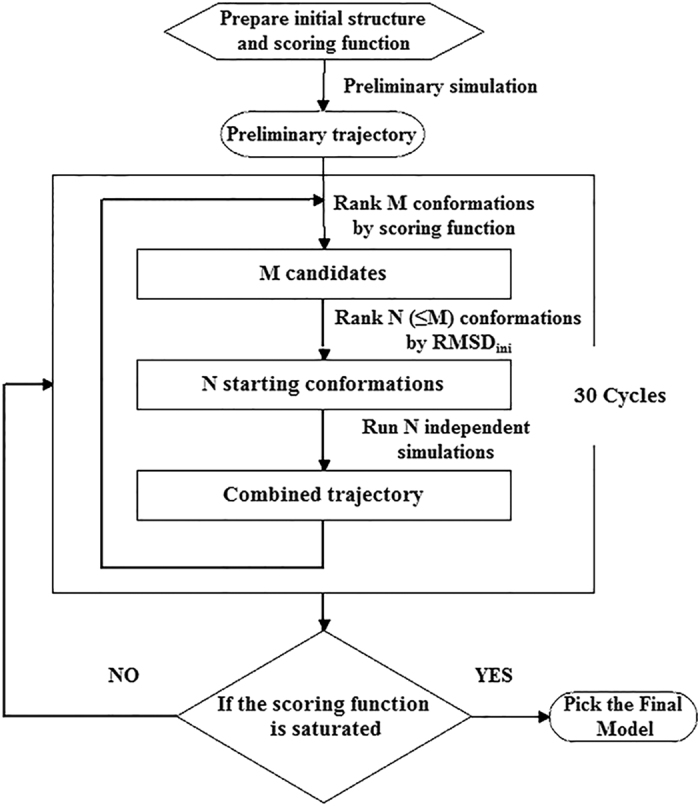
General work flow of PaCS-Fit. After preparation of the initial structure, a preliminary simulation is carried out. 30 cycles of parallel simulations are considered as a unit. Once the scoring function is saturated, the simulations can be stopped. Otherwise, PaCS-Fit would run for another 30 cycles. The final structural model is picked at the cycle where the scoring function starts to saturate.

The PaCS-Fit method consists of the following steps (Fig. 6):

1. Obtain an initial conformation and the target low-resolution structural data of the biomolecule. The initial conformation could be an experimental structure or a structural model. A scoring function is designed according to the type of the low-resolution data.
2. Starting from the initial conformation, a preliminary simulation is carried out. Any simulation method and software package can be utilized by PaCS-Fit with no need of changing the code, such as atomistic MD simulations, enhanced sampling techniques, or coarse-grained modeling.
3. For each simulated conformation, the scoring function, as well as RMSD_ini_, is calculated. For the benchmark systems, RMSD_tar_ were also computed. Only Cα atoms were used to measure these RMSD values. From the *M* conformations that best fit the target low-resolution data, *N* out of them, which have the largest RMSD_ini_ values, are chosen. For all the proteins studied in this paper, we set *M* = 20 and *N* = 10. The rationale to select *M* and *N* is discussed in the Supplementary Information.
4. Starting from the *N* selected conformations, independent simulations are run. Again one can choose various simulation methods and software. It has been recognized that multiple independent short-time simulations have better sampling efficiency than a single long-time simulation^52^.
5. Repeat 3 and 4 for 30 cycles. If the scoring function is saturated, we stop the simulation and then pick the conformation at the cycle that starts to saturate as the final structural model. Otherwise, we run PaCS-Fit for another 30 cycles.

### Simulation methods and software

Simulations carried out in this paper were generally standard MD but sometimes an enhanced sampling method called amplified collective motions (ACM)^53^, using the GROMACS-4.5.5 package^54^. As in the original PaCS-MD method^21^,^22^, the preliminary simulation of any other protein except for the GroEL monomer was a 0.1-ns MD. After that, for all the proteins, every cycle of PaCS-Fit included ten independent 0.1-ns MD simulations. The GroEL monomer has 525 residues that is the largest protein we studied, it would take considerable CPU time to do PaCS-Fit using only conventional MD simulations. Therefore, in both the close-to-open and open-to-close PaCS-Fit, we carried out a 2-ns preliminary ACM simulation for the GroEL monomer, in order to quickly obtain conformations with larger CC to the target EM map than those from only a 0.1-ns MD. After the enhanced sampling, the following cycles switched to standard MD simulations to make the protein further approach the target data gradually. Setup details of all the simulations are described in the Supplementary Information.

### Scoring functions

PaCS-Fit can use any low-resolution structural data conveniently, as long as a scoring function is properly designed. In this paper, we have implemented two types of low-resolution data, which are from SAXS and EM, respectively.

#### SAXS

For any conformation, its SAXS profile can be computed by programs like CRYSOL^46^ in the ATSAS package^55^. Then the discrepancy between the theoretical SAXS profile and the target SAXS data is calculated as


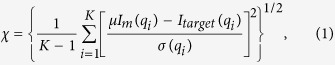


where *K* is the number of data points in *I*_*target*_(*q*), and *σ*(*q*) are standard deviations. *I*_*m*_(*q*) is the SAXS profile of the simulated conformation, and *μ* is a scaling factor. *q* = 4*π*sin *θ*/*λ* is the momentum transfer where 2*θ* is the scattering angle and λ is the wavelength.

#### EM

The cross-correlation coefficient (CC) between the simulated map of any sampled conformation and the target EM map is computed as


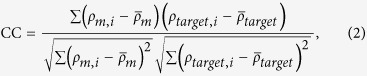


where *ρ*_*m,i*_ is the electron density of simulated map at the *i*^th^ voxel and *ρ*_*target,i*_ is the corresponding density in the target map. During PaCS-Fit, all the CC values were computed by the collage program^47^ in the Situs package^36^.

### Simulated SAXS data of AKeco

The target SAXS data of AKeco was simulated as follows. From a 0.1-ns MD simulation of the target structure, theoretical SAXS curves of *L*=100 conformations in the trajectory were computed by the program CRYSOL^46^, respectively. The simulated SAXS profile of the target state was then obtained by taking the average


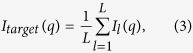


where *I*_*l*_(*q*) is the theoretical SAXS curve of the *l*^*th*^ conformation. *σ*(*q*) in equation (1) was calculated as the standard deviation of the 100 SAXS curves.

### Initial conformation of HEWL in SAXS-targeted PaCS-Fit

For HEWL, a 1-ns ACM simulation was performed, starting from its crystal structure. In the trajectory, the conformation with the largest RMSD of 6.2 Å to the crystal structure was chosen as the initial conformation of the PaCS-Fit. The simulation details are described in the Supplementary Information.

## Additional Information

**How to cite this article**: Peng, J. and Zhang, Z. Unraveling low-resolution structural data of large biomolecules by constructing atomic models with experiment-targeted parallel cascade selection simulations. *Sci. Rep.* **6**, 29360; doi: 10.1038/srep29360 (2016).

## Supplementary Material

Supplementary Information
