# Supplementary material for: Unraveling low-resolution structural data of large biomolecules by constructing atomic models with experiment-targeted parallel cascade selection simulations
Source: Sci Rep. 2016 Jul 5;6:29360. doi: 10.1038/srep29360 (PMC4932515; doi:10.1038/srep29360)
Supplement: Supplementary Information [file srep29360-s1.pdf]

Supplementary Information: Unraveling low-resolution structural data of large biomolecules by constructing atomic models with experiment-targeted parallel cascade selection simulations

Junhui Peng and Zhiyong Zhang*

Hefei National Laboratory for Physical Science at Microscale and School of Life Sciences, University of Science and Technology of China, Hefei, Anhui 230026, People's Republic of China

*Corresponding author: Zhiyong Zhang, Tel: +86-551-63600854; Fax: +86-551-3600374; Email: zzyzhang@ustc.edu.cn

Criteria to pick the final structural model

Several possible criteria, including the scoring function, $RMSD_{ini}$ and R_g, are investigated. In the close-to-open fitting of AKeco targeted by the SAXS data, we plotted all these values at each cycle, along with the $RMSD_{tar}$ values (Fig. S1).

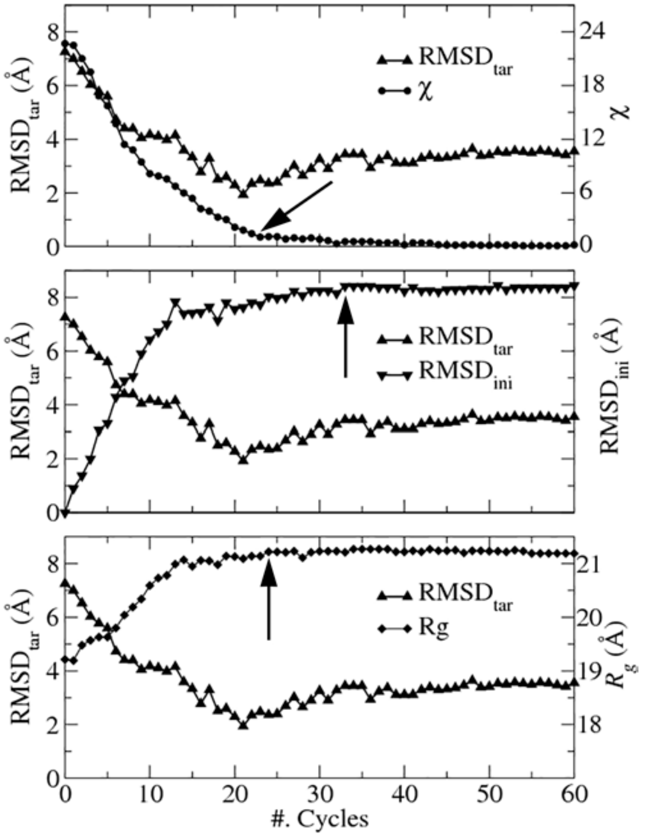

Figure S1. Possible criteria to pick the final structure model in PaCS-Fit. (a) The minimal χ to the simulated target SAXS profile at each cycle (circles). The final structural model was chosen at the 23rd cycle (indicated by an arrow). (b) $RMSD_{ini}$ (down-triangles). The final model was picked at the 33rd cycle. (c) R_g (squares). The final model was chosen at the 24th cycle. In each panel, the corresponding $RMSD_{tar}$ to the open structure are also shown (up-triangles).

If we select the final structural model according to the saturation of the scoring function χ (Fig. S1a, circles), the conformation at the 23rd cycle would be picked that has a $RMSD_{tar}$ of 2.5 Å. However, the saturation point of $RMSD_{ini}$ is at the 33rd cycle (Fig. S1b, down-triangles), and

the corresponding conformation has a $RMSD_{tar}$ of 3.5 Å. After the 24th cycle, R_g becomes saturated at about 21.2 Å (Fig. S1c, squares) that is consistent to the value (21.2±0.1 Å) estimated from the target SAXS data by Guinier analysis, and the conformation at this point has a $RMSD_{tar}$ of 2.4 Å. It seems that either χ or R_g could serve as a criterion here to pick the final structural model. Considering that R_g might not work well when integrating other types of low-resolution data in PaCS-Fit, we finally use the saturation of the scoring function as the criterion since it looks like the most straightforward way to pick the final model.

Estimation of model precision

In a real application of PaCS-Fit, the target structure of the protein is unknown, so one cannot be certain of the accuracy of the structural model. However, model precision and accuracy may be estimated based on variability of multiple built models[1]. We have addressed this issue on HEWL and the triple-BRCT-domain of ECT2, respectively.

For HEWL, we ran ten independent PaCS-Fit targeted by the SAXS data, from the same initial conformation. All the χ values are saturated at about 0.4. R_g of the ten structural models are from 15.0 to 15.1 Å, which are close to the value (15.3±0.2 Å) estimated from the SAXS data. Pretending no knowledge of the target crystal structure, we have calculated the pairwise RMSD values among these models, which are from 1.1 to 2.2 Å with the mean value of 1.5 Å. The above data suggest that the structural models of HEWL built by PaCS-Fit are reliable with high precision, and the variability of them may provide a lower bound of the model accuracy[1].

For the triple-BRCT-domain of ECT2, ten independent SAXS-targeted PaCS-Fit were also carried out, starting from its crystal structure. Although their χ values are all fairly small from 0.4

to 0.6, and R_g (from 26.6 to 27.7 Å) are all similar to the value (27.5±0.4 Å) estimated from the experimental SAXS data, the ten structural models demonstrate some different conformations with pairwise RMSD values ranging from 2.1 to 8.3 Å. The results may indicate that the SAXS data alone is insufficient to determine a single model of this protein or there exist multiple conformations[1]. Therefore, it seems unlikely to estimate the model precision here unless more experimental data would be available.

Cross validation of the PaCS-Fit models

One type of analysis to indicate the model accuracy is cross validation, that is, the structural model is built by one set of experimental data and then validated against other data sets not included in the integrative modeling[1]. For AKeco, both the simulated SAXS and EM data are available, which enable us to do cross validation (Table S1). In the close-to-open fitting of the protein, the atomic model constructed by SAXS-targeted PaCS-Fit has a χ value of 1.0, and its CC=0.87. The model obtained by EM-targeted PaCS-Fit has a CC value of 0.94, and its χ equals to 5.3 that is fairly small (Fig. 1a, circles). In the open-to-close fitting of AKeco, the χ value of the structural model built by SAXS-targeted PaCS-Fit is 3.8, and this model has a CC=0.84. The model from EM-targeted PaCS-Fit has a CC value of 0.92, and χ is as small as 2.7. The above cross validation may suggest that these PaCS-Fit models of AKeco are reliable, which are supported by their $RMSD_{tar}$ values (Table S1). Generally the CC values are consistent to the $RMSD_{tar}$ values, that is, the larger CC the smaller $RMSD_{tar}$. However, this may not be the case between χ and $RMSD_{tar}$ because the SAXS data has a lower resolution than the EM data that may lead to an overfitting problem.

Direction	Target data	χ	CC	$RMSD_{tar}$ (Å)
close-to-open	SAXS	**1.0**	*0.87*	2.5
	EM	*5.3*	**0.94**	1.5
open-to-close	SAXS	**3.8**	*0.84*	2.9
	EM	*2.7*	**0.92**	2.1

Table S1. Cross validation of the AKeco models generated by PaCS-Fit. These numbers in **bold** were scores used in PaCS-Fit to pick the final structural model, and those in *italic* were back-calculated from the selected model.

PaCS-Fit targeted by the SAXS and EM data simultaneously

We have also tried a close-to-open fitting of AKeco targeted by the SAXS and EM data simultaneously, which can be easily implemented in our PaCS-Fit tool. After each cycle of the simulations, 40 conformations with the largest CC against the target EM map were selected from the trajectory, then 20 out of 40 conformations were chosen with the smallest χ values against the target SAXS data, and finally 10 out of 20 conformations with the largest $RMSD_{ini}$ values were picked out to start the next cycle. The results of this EM+SAXS-targeted PaCS-Fit are shown in Figure S2. Both CC and χ become saturated after the 24th cycle, so we pick the conformation at this point as the final structural model with χ=2.1 and CC=0.93. The $RMSD_{tar}$ of this model is only 1.3 Å. The results suggest that by combining both SAXS and EM data, we can also obtain reliable structural models of AKeco.

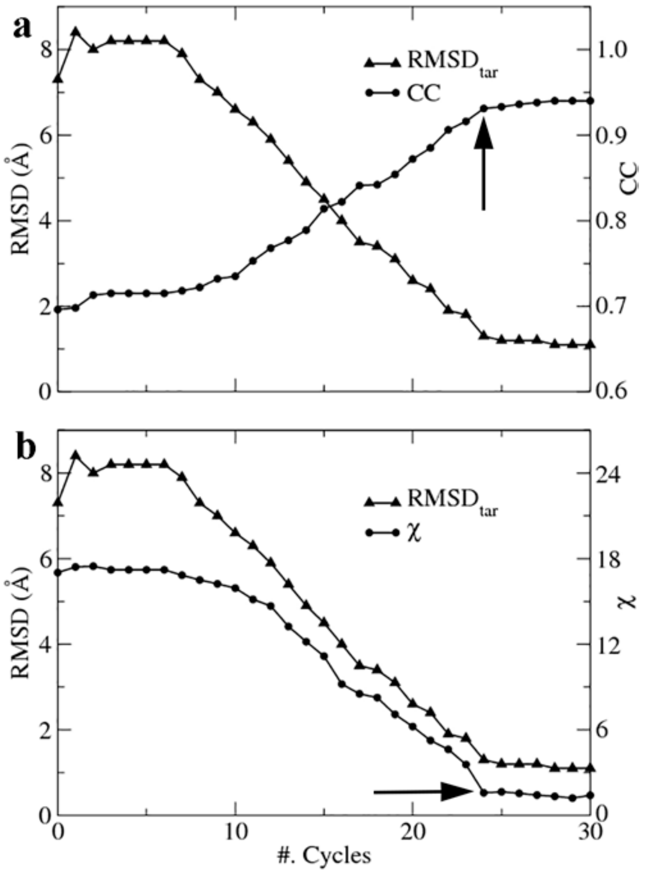

Figure S2. EM+SAXS-targeted PaCS-Fit of AKeco from the closed to the open state. (a) The maximal CC value to the simulated target EM map at each cycle (circles), and the corresponding $RMSD_{tar}$ (up-triangles). (b) The minimal χ to the simulated target SAXS profile at each cycle (circles), and the corresponding $RMSD_{tar}$ (up-triangles). The final structural model was chosen at the 24th cycle (indicated by an arrow) since both CC and χ become saturated after this point.

Selection of *M* and *N* in PaCS-Fit

In all of the PaCS-Fit, we ran N=10 independent MD simulations at each cycle after the preliminary simulation, as in the original PaCS-MD method[2,3]. For any of these proteins, ten independent MD simulations have already achieved efficient sampling to explore its conformational space. If more computational resources are available, one could try a larger N, with which fewer cycles may be needed to fit the low-resolution structural data. However, we can still set N=10, and allocate more cores to run individual MD simulation. In the latter case,

although more cycles may be necessary, each PaCS-Fit cycle would be done faster than that in the former case.

Besides *N*, PaCS-Fit has an additional parameter *M*, that is, at each cycle, *M* conformations that best fit the low-resolution structural data are selected from the trajectory, and *N* out of *M* conformations that have the largest $RMSD_{ini}$ are used to start the next cycle. In the original PaCS-MD method[2], the 'scoring function' is $RMSD_{tar}$, whereas in PaCS-Fit, the scoring function is from the low-resolution data that may provide a weaker restraint towards the target than $RMSD_{tar}$. Therefore, $RMSD_{ini}$ is used to encourage the protein to escape from its initial state. *M* should be larger than *N*. When M=N, we did observe the protein conformation was sometimes 'trapped' and could not transit to the target. On the other hand, *M* cannot be too larger than *N*, otherwise the major driving force in PaCS-Fit would be $RMSD_{ini}$ instead of the low-resolution structural data. We have found that M=20 seems appropriate with N=10.

When using the PaCS-Fit method, we suggest users to try M=20 and N=10 as well, for their own problems, which should work well in most cases.

Simulation details

AKeco

MD. Standard MD simulations were used in both the SAXS- and EM-targeted PaCS-Fit of AKeco, which were carried out using the GROMACS-4.5.5 package[4] and the AMBER03 force field[5]. The setup procedure was as follows. The periodic boundary condition (PBC) with a dodecahedron box type was used, with the minimum distance between the solute and the box boundary of 1.2 nm. The box was filled with TIP3P water molecules[6]. The system with the

protein and waters was energy-minimized by the steepest descent method, until the maximum force was smaller than 1000 kJ mol^{-1} nm^{-1}. 4 Na^+ were added to compensate the net negative charges on the protein by replacing the same number of water molecules with the most favorable electrostatic potential. The final system was energy-minimized again using the steepest descent and then the conjugate gradient method, until the maximum force was smaller than 100 kJ mol^{-1} nm^{-1}. The simulation was conducted by using the leap-frog algorithm[7] with a 2 fs time step. Before the production run, a 100 ps equilibration simulation with positional restraint was carried out, using a force constant of 1000 kJ mol^{-1} nm^{-2}. The initial atomic velocities were generated according to a Maxwell distribution at 300 K. The simulation was performed under the constant NPT condition. The three groups (protein, solvent, and ions) were coupled separately to a temperature bath of 300 K by using an velocity rescaling thermostat[8], with a relaxation time of 0.1 ps. The pressure was kept at 1 bar with a relaxation time of 0.5 ps and the compressibility of 4.5×10^{-5} bar^{-1}. Covalent bonds in the protein were constrained using the P-LINCS algorithm[9]. Twin-range cutoff distances for the van der Waals interactions were chosen to be 0.9 and 1.4 nm, respectively, and the neighbor list was updated every 20 fs. The long-range electrostatic interactions were treated by the PME algorithm[10], with a tolerance of 10^{-5} and an interpolation order of 4.

HEWL

MD. Standard MD simulations were used in the SAXS-targeted PaCS-Fit of HEWL. The setup procedure was much the same as that of AKeco, except that 8 CL^- were added to the system to compensate the net positive charges on the protein.

ACM. This enhanced sampling method was used to generate an initial conformation for SAXS-targeted PaCS-Fit of HEWL. ACM[11] has been implemented in the GROMACS-4.5.5 package[4]. Accelerated sampling was started from the conformation after the equilibration simulation. Many parameters were the same as those in the standard MD simulation, except that collective motions described by an elastic network model (ENM)[12] were amplified by coupling them to a high-temperature bath. From an all-atom structure of the protein in the simulation, an ENM was built with CG sites locating at the center-of-mass (COM) of residues. The potential energy function of ENM takes a harmonic form

$$V = \sum_{i,j>i} \frac{1}{2} k_{ij} \Delta r_{ij}^2 , \tag{S1}$$

where Δr_{ij} is the fluctuation of the COM distance between residues i and j, and k_{ij} is the spring constant,

$$k_{ij} = \begin{cases} 1.0 & r_{ij} \le 0.7\ nm \\ 10^{-2} & 0.7 < r_{ij} \le 1.1\ nm \\ 5\times 10^{-4} & 1.1 < r_{ij} \le 1.4\ nm \\ 0 & r_{ij} > 1.4\ nm \end{cases} . \tag{S2}$$

Three cut-off distances, 0.7, 1.1, and 1.4 nm, respectively, were used to model interactions at different ranges.

A Hessian matrix of the second derivatives of the overall potential (Eqn. S1) was constructed, and then diagonalized to yield a matrix of eigenvectors and corresponding eigenvalues. Each eigenvector with a non-zero eigenvalue is called a normal mode, and the corresponding eigenvalue is the frequency. Usually only a few ENM modes with the lowest frequencies are

dominant in collective motions of the protein. For HEWL, we took the six slowest modes to define an essential subspace. At each time step, the velocity of each atom was divided into two parts, which were the projection onto the essential subspace and the rest, respectively. By modifying the weak coupling method[13], the component of velocity in the essential subspace was coupled to a high temperature of 1000 K while the remaining velocity was coupled normally to 300 K, and finally the updated velocity was the combination of the two components. During the ACM simulation, collective modes were updated on the fly by doing ENM calculation every 100 time steps according to the new generated protein conformation.

Triple-BRCT-domain of ECT2

MD. Standard MD simulations were used in the SAXS-targeted PaCS-Fit of the triple-BRCT-domain of ECT2. The setup procedure was much the same as that of AKeco, except for the following. 137 Na^+ and 125 CL^- were added to the system to not only compensate the net negative charges on the protein, but also mimic salt concentration (0.15 M) of the protein sample for SAXS experiments. The initial velocities of the system were generated at 310 K, and the temperature bath was set to 310 K as well.

GroEL monomer

MD. For each EM-targeted PaCS-Fit of the GroEL monomer, standard MD simulations were used to run these cycles after the preliminary simulation. The simulation parameters were the same as that of AKeco, except for the following. When setting up MD staring from the closed structure, the minimum distance between the solute and the box boundary was 2.0 nm to assure that the box may have enough space to allow the close conformation to transit to the open state

during the PaCS-Fit. 19 Na^+ were added in order to compensate the net negative charges on the protein.

ACM. The enhanced sampling method was used to run the preliminary simulation in each EM-targeted PaCS-Fit of the GroEL monomer. The parameters of ACM were largely the same as those for HEWL, except for the following. The four slowest modes were used to define an essential subspace. At each time step, the component of velocity in the essential subspace was coupled to a high temperature of 900 K while the remaining was coupled to 300 K. During the ACM simulation, collective modes were updated every 500 time steps.

Computational cost of PaCS-Fit

In this work, we used either 80 Intel cores (2.6 GHz) or 160 AMD cores (2.3 GHz) to run PaCS-Fit (Table S2). The computational cost mainly consists of a preliminary MD or ACM simulation, ten independent MD simulations at each cycle, and calculations of scoring functions for all the simulated conformations. It should be noted that, for the same protein, computation of CC is more expensive than that of χ.

Proteins	Target data	System size (# of atoms)	# of cores	Total time scale	Total CPU time (min)
AKeco-close	SAXS	32916	80	0.1+60	684
AKeco-open		45642	80	0.1+60	894
HEWL		26286	80	0.1+30	280
Triple-BRCT-domain of ECT2		108281	80	0.1+30	886
AKeco-close	EM	32916	80	0.1+30	483
AKeco-open		45642	80	0.1+60	1177
GroEL monomer-close		127720	160	2+30	3234
GroEL monomer-open		98849	160	2+60	5382

Table S2. Computational cost of PaCS-Fit. The total time scale of each PaCS-Fit includes a preliminary simulation (0.1-ns MD or 2-ns ACM) followed by 30 or 60 cycles of ten independent 0.1-ns MD simulations.

References

1 Schneidman-Duhovny, D., Pellarin, R. & Sali, A. Uncertainty in integrative structural modeling. *Curr. Opin. Struc. Biol.* **28**, 96-104, (2014).

2 Harada, R. & Kitao, A. Parallel cascade selection molecular dynamics (PaCS-MD) to generate conformational transition pathway. *J. Chem. Phys.* **139**, (2013).

3 Harada, R. & Kitao, A. Nontargeted parallel cascade selection molecular dynamics for enhancing the conformational sampling of proteins. *J. Chem. Theory Comput.* **11**, 5493-5502, (2015).

4 Hess, B., Kutzner, C., van der Spoel, D. & Lindahl, E. GROMACS 4: algorithms for highly efficient, load-balanced, and scalable molecular simulation. *J. Chem. Theory. Comput.* **4**, 435-447, (2008).

5 Duan, Y. *et al.* A point-charge force field for molecular mechanics simulations of proteins based on condensed-phase quantum mechanical calculations. *J. Comput. Chem.* **24**, 1999-2012, (2003).

6 Jorgensen, W. L., Chandrasekhar, J., Madura, J. D., Impey, R. W. & Klein, M. L. Comparison of simple potential functions for simulating liquid water. *J. Chem. Phys.* **79**, 926-935, (1983).

7 Hockney, R. W., Goel, S. P. & Eastwood, J. W. Quiet high-resolution computer models of a plasma. *J. Comput. Phys.* **14**, 148-158, (1974).

8 Bussi, G., Donadio, D. & Parrinello, M. Canonical sampling through velocity rescaling. *J. Chem. Phys.* **126**, (2007).

9 Hess, B. P-LINCS: a parallel linear constraint solver for molecular simulation. *J. Chem. Theory. Comput.* **4**, 116-122, (2008).

10 Essmann, U. *et al.* A smooth particle mesh Ewald method. *J. Chem. Phys.* **103**, 8577-8593, (1995).

11 Zhang, Z. Y., Shi, Y. Y. & Liu, H. Y. Molecular dynamics simulations of peptides and proteins with amplified collective motions. *Biophys. J.* **84**, 3583-3593, (2003).

12 Atilgan, A. R. *et al.* Anisotropy of fluctuation dynamics of proteins with an elastic network model. *Biophys. J.* **80**, 505-515, (2001).

13 Berendsen, H. J. C., Postma, J. P. M., Vangunsteren, W. F., Dinola, A. & Haak, J. R. Molecular dynamics with coupling to an external bath. *J. Chem. Phys.* **81**, 3684-3690, (1984).
